# Antimicrobial activity of tetraacetylethylenediamine-sodium perborate versus sodium hypochlorite against *Enterococcus faecalis*

**DOI:** 10.15171/joddd.2016.007

**Published:** 2016-03-16

**Authors:** Sahar Shakouie, Amin Salem Milani, Mahsa Eskandarnejad, Saeed Rahimi, Mohammad Froughreyhani, Saeede Galedar, Ehsan Ranjbar

**Affiliations:** ^1^Assistant Professor, Department of Endodontics, Faculty of Dentistry, Tabriz University of Medical Sciences, Tabriz, Iran; ^2^Assistant Professor, Dental and Periodontal Research Center, Tabriz University of Medical Sciences, Tabriz, Iran; ^3^Professor, Department of Endodontics, Faculty of Dentistry, Tabriz University of Medical Sciences, Tabriz, Iran; ^4^Associate Professor, Department of Endodontics, Faculty of Dentistry, Tabriz University of Medical Sciences, Tabriz, Iran; ^5^Post-graduate Student, Department of Endodontics, Faculty of Dentistry, Tabriz University of Medical Sciences, Tabriz, Iran; ^6^Dentist, Private Practice, Tabriz, Iran

**Keywords:** Disk diffusion antimicrobial test, Enterococcus faecalis, sodium hypochlorite, tetraacetylethylenediamine

## Abstract

***Background. ***This study evaluated the antimicrobial activity of Tetraacetylethylenediamine-sodium perborate (TAED-SP) in comparison to 2.5% and 5% sodium hypochlorite (NaOCl) against Enterococcus faecalis.

***Methods.*** A standard suspension of E. faecalis was inoculated on 60 plates containing Mueller-Hinton agar culture medium. Four sterile disks of Beckman filtration paper were placed on each plate. TAED-SP, 5% and 2.5% NaOCl were placed on three disks. Sterile physiologic saline was placed on the fourth disk as negative control. After 24-hour incubation, the diameter of the inhibition zone around the disks was measured using a transparent ruler. One-way Analysis of Variance (ANOVA) was used to compare the mean zone of microbial growth in the groups. P-values less than 0.05 were considered statistically significant.

***Results.*** There was a significant difference in the diameter of the inhibition zones between groups (P < 0.05). The Tukey post hoc test showed a higher diameter of the inhibitory zone with TAED-SP than that of 2.5% NaOCl. However, there were no significant differences between the inhibitory zones of TAED-SP and 5% NaOCl.

***Conclusion. ***TAED-SP and 5% NaOCl have similar antibacterial activity against E. faecalis; however, TAED-SP has a greater antibacterial effect compared to 2.5% NaOCl.

## Introduction

Microorganisms are the main etiologic agents for development of pulp and periapical diseases.^1^ Therefore, the principal aim of root canal therapy is the elimination of necrotic tissues and microorganisms from the root canal system and prevention of recontamination of the pulp space after treatment. Failure of endodontic treatments is attributed to the incomplete elimination of bacteria from infected canals or penetration of oral bacteria into the root canal system during treatment, between appointments, or after completion of the treatment.^2,3^

Mechanical debridement is a critical stage in endodontic treatment; however, it alone cannot completely eliminate microorganisms from inaccessible areas. Therefore, different irrigants with antimicrobial activities are available in order to improve and complement mechanical debridement of the root canal system.^4-6^ An ideal endodontic irrigating solution should be a highly effective disinfectant, offer antibacterial substantivity, able to dissolve pulp tissue and inactivate endotoxins, be nonantigenic, nontoxic, and noncarcinogenic , have no adverse effects on dentin, be convenient to apply, and cause no tooth discoloration.^7-9^ At present, sodium hypochlorite (NaOCl) is the most commonly used root canal irrigant. It has adequate tissue solubility and antimicrobial effect.^10^ However, it has unfavorable taste and odor, and its toxic and caustic effects on vital tissues resulting in hemolysis, and necrosis are still a matter of concern.^11^ NaOCl also has been shown to deplete dentin of organic compounds and to increase the permeability of dentin significantly.^12^ It does not effectively wet dentin, and small canals and canal extensions are poorly irrigated.^13^ Sodium hypochlorite has also detrimental corrosive effect on metals and some preparation instruments.^14^ Considering the adverse effects of NaOCl, an antibacterial agent with similar antibacterial activity but without adverse effects of NaOCl may be appropriate to substitute NaOCl.^15^ Tetraacetylethylenediamine (TAED) is a material produced by acetylating the ethylenediamine. It has no color and odor and is stable and easy to use. It is a non-toxic, non-allergenic, and non-mutagenic material. TAED is an industrial detergent with a bleaching activity, which can apply its bleaching effect at lower temperatures compared to other materials.^16^ TAED has less cytotoxicity than NaOCl.^17^ Spratt et al^18^ showed that mixture of TAED and sodium perborate (SP) provided effective control on the dental unit water line biofilms. This antibacterial activity against biofilms is attributed to the release of oxygen radicals from TAED-SP.^18^ The release of free radicals may make TAED-SP a potentially effective antimicrobial irrigant for endodontic use. However, the antibacterial effect of TAED-SP on endodontic pathogens has not been studied yet. Therefore, this study was conducted to assess the antibacterial effect of TAED-SP against *Enterococcus faecalis* as a resistant endodontic bacterium in comparison with NaOCl.

## Methods

This was an experimental study on bacteria without involving animal or human subjects, and therefore, an ethical approval was not required.

Sixty Muller-Hinton agar culture plates (Merck, Germany) were used in this study. The plates were incubated at 37ºC under aerobic conditions for 24 hours in order to confirm their sterility. E. faecalis (American type culture collection [ATCC] 29212) was obtained and maintained in brain-heart infusion (BHI) broth. The density of inoculum was adjusted to the turbidity of 0.5 McFarland (1.5 × 108 bacteria/ml). The bacteria were seeded on agar plates.

**Figure 1.**
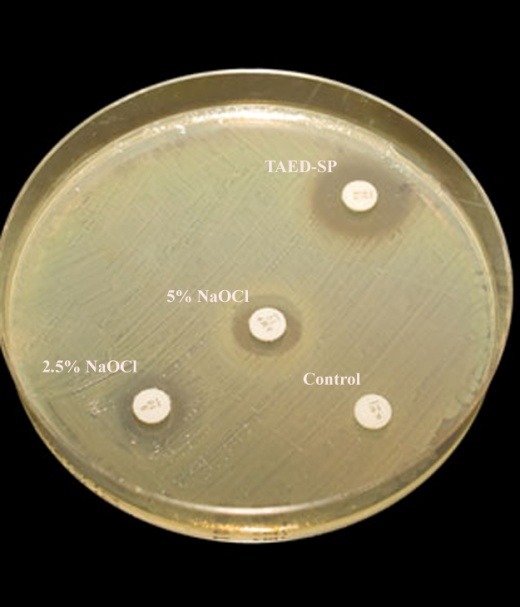
Agar diffusion test.

Three irrigation solutions were evaluated in the present study: TAED-SP (Jahad, Tehran, Iran), 2.5% and 5% NaOCl (Merck, Darmstadt, Germany). Ten percent TAED-SP solution was prepared by mixing TAED with SP with a ratio of 2:8. The mixture was then diluted with distilled water to a ratio of 1:10. NaOCl 2.5% solution used in this study was prepared by diluting 5% NaOCl by sterile distilled water without any preservative. Four sterile disks of Beckman filter paper, with a diameter of 6 mm, were placed on the culture media (Figure 1). Ten microliter of each solution was placed on separate disks, and 10 μL sterile physiologic saline was placed on the fourth disk as negative control. The plates were incubated aerobically at 37º C for 48 hours. After incubation, the zone of bacterial inhibition around each disk was measured by a blind examiner as the shortest distance (mm) from the outer margin of the disks to the initial point of bacterial growth (Figure 1). All the procedures were carried out under aseptic condition.

### Statistical analysis 

Statistical analysis was performed by SPSS for windows (SPSS Inc, Chicago, IL). Normality of data was assessed by Kolmogorov-Smirnov test. One-way Analysis of Variance (ANOVA) followed by Tukey post hoc test was used to compare the mean zone of microbial growth in the groups. P-values less than 0.05 were considered statistically significant.

## Results

The mean and standard deviation of the diameters of microbial inhibitory zones have been presented in Table 1. No zone of inhibition was observed adjacent to negative control disks. Kolmogorov-Smirnov test confirmed normal distribution of data (Z = 1.34, P = 0.054). Statistical analysis showed significant differences between the groups (P = 0.00). The mean diameter of microbial inhibitory zone of TAED-SP was greater than that of 2.5% NaOCl (P < 0.05); however, there were no significant differences in the mean diameters of inhibitory zone of 5% NaOCl and TAED-SP or 2.5% NaOCl (P > 0.05).

**Table 1:** Mean zone of microbial growth inhibition (mm) produced by test irrigants against enterococcus faecalis

**Groups**	**Mean (±SD)**	**Min**	**Max**
**TAED-SP**	7.9 (±1)^a^*^^	6	9
**2.5% NaOCl**	6.9 (±1)^b^	5	8
**5% NaOCl**	7.7 (±1)^ab^	6	9

SD: standard deviation; TAED-SP: Tetraacetylethylenediamine-sodium perborate; NaOCl: Sodium hypochlorite; ^*^different letters denote statis-tically significant difference

## Discussion

In the present study, standard microbiologic techniques were used to determine the antimicrobial activity of TAED-SP in comparison with NaOCl in an in vitro model. Combination of TAED and SP was used in this study because, according to Spratt et al,^18^ mixing TAED with SP accelerates release of free radicals in low temperature. Agar diffusion test is a simple, inexpensive, and well-standardized method of antibacterial testing.^19^ This method is especially appropriate for determining the antimicrobial ability of water-soluble materials. A variety of materials may be tested quickly using this method including liquids, solid materials and coated antimicrobial surfaces.^19,20^ The bacterial species used in this study was E. faecalis because it is the predominant bacterial species in resistant endodontic infections.^21,22^ It colonizes in dentinal tubules and penetrates into the entire width of dentin.^23^ E. faecalis is easy to culture and it grows rapidly, and has been widely used in endodontic in vitro studies.^20,22,24-26^

This study showed that TAED-SP and 5% NaOCl have similar antibacterial activity which is stronger than 2.5% NaOCl. This result confirms the weak antibacterial potency of diluted NaOCl solutions shown in previous studies.^26,27^ The antibacterial activity of TAED-SP has also been shown in other studies. Spratt et al. showed that TAED-SP was completely effective in controlling the biofilms in the dental unit waterlines contaminated with Streptococcus oralis, *E. faecalis*, and *S. aureus*.^18^ A study by Puttaiah et al^28^ showed that contact with TAED-SP and perborate eliminated all the vegetative microorganisms in 30 seconds and bacterial spores in 5 minutes.

As stated earlier, cytotoxicity of irrigants is another important issue because they come in contact with periradicular tissues during endodontic treatment, and their toxicity results in tissue damage and interferes with regenerative process.^14^ Therefore, in selecting an ideal endodontic irrigant, a material with profound antimicrobial effect and minimum cytotoxicity is desirable. Based on the results of the study by Simbola et al,^17^ a mixture of TAED and SP is less toxic than NaOCl solution of similar dosage. Therefore, TAED-SP may be an acceptable alternative to NaOCl, if the future studies reveal other advantages of TAED-SP over NaOCl.

The results of this study should be interpreted with caution. Despite numerous advantages, agar diffusion test has some drawbacks. Inhibition zones do not always have clear or regular boundaries and are influenced by the diffusion rate of materials through the agar.^20,29,30^ Furthermore, determining antibacterial activity by this method may not reflect the *in vivo* condition where bacteria grow as biofilm on complex root canal surfaces. Therefore, further studies are necessary to determine the antibacterial activity of TAED-SP against bacterial biofilms on root canal dentin.

## Conclusion

Within the limitations of this in vitro study, we concluded that TAED-SP and 5% NaOCl have similar antibacterial activity against E. faecalis; however, TAED-SP has a greater antibacterial effect compared to 2.5% NaOCl.

## Acknowledgments

The authors would like to thank Dental and Periodontal Research Center at Tabriz University of Medical Sciences for financial support of this research project. 

## Authors’ contributions

The study concept was developed by SS, ME, SR, and MF who also contributed to the study proposal. SG and ER conducted the study. SS supervised SG and ER in conducting the study experiments. ASM drafted the manuscript. All authors had contributions in critically revising the manuscript. All authors have read and approved the final manuscript.

## Funding

The Dental and Periodontal Research Center at Tabriz University of Medical Sciences financially supported the study.

## Competing interests

Dr. Saeed Rahimi is a member of the editorial board of the *Journal of Dental Research, Dental Clinics, Dental Prospects*. The authors have no other competing interests to declare with regards to authorship and/or publication of this article.

## Ethics approval

Not applicable.
